# Does premedication with dexmedetomidine provide perioperative hemodynamic stability in hypertensive patients?

**DOI:** 10.1186/1471-2253-14-113

**Published:** 2014-12-10

**Authors:** Gulbin Sezen, Yavuz Demiraran, Ilknur Suidiye Seker, Ibrahim Karagoz, Abdulkadir Iskender, Handan Ankarali, Ozlem Ersoy, Onur Ozlu

**Affiliations:** Department of Anesthesiology and Reanimation, Duzce University Faculty of Medicine, Duzce, Turkey; Department of Biostatistics, Duzce University Faculty of Medicine, Duzce, Turkey

**Keywords:** Dexmedetomidine, Midazolam, Premedication, Hypertension

## Abstract

**Background:**

Perioperative hemodynamic fluctuations are seen more often in hypertensive patients than in normotensive patients. The purpose of our study was to investigate the perioperative hemodynamic effects of dexmedetomidine and midazolam used for premedication in hypertensive patients relative to each other and in comparison to normotensive patients.

**Methods:**

One-hundred-forty female, normotensive or hypertensive patients undergoing myomectomies or hysterectomies. They were randomly enrolled into the subgroups: Group ND (normotensive-dexmedetomidine); Group HD (hypertensive-dexmedetomine); Group NM (normotensive-midazolam); Group HM (hypertensive- midazolam). Dexmedetomidine was administered at a concentration of 0.5 μg.kg^−1^, and midazolam was administered at a concentration of 0.025 μg.kg^−1^ via intravenous (IV) infusion before the induction of anaesthesia. Haemodynamic parameters were recorded at several times (T_beginning_, T_preop5 min_, T_preop 10 min_, T_induction_, T_intubation_, T_intubation__5 min_, T_initial surgery_, T_surgery 15 min_, T_surgery 30 min_, T_extubation_, T_extubation 5 min_). Propofol amount for induction, time between induction and initial surgery, demand of antihypertensive therapy, rescue atropine were recorded. Quantitative clinical and demographic characteristics were compared using One Way ANOVA. The values were compared using One-way Analysis of Variance. Additionally periodic variations were examined by One way Repeated Measures Analysis of Variance for groups separately.

**Results:**

SBP was significantly different between normotensive and hypertensive groups at the following time points: T_preop 5 min_, T_preop 10 min_, T_induction_, T_intubation_*,* T_intubation 5 min_ and T_initial surgery_. MBP was significantly different in the hypertensive groups at T_induction_, T_intubation_, T_intubation 5 min,_ T_initial surgery_, T_surgery 15 min_, T_surgery 30 min,_ T_extubation_ and T_extubation 5 min_. The perioperative requirements for antihypertensive drugs were significantly higher in Group HM.

**Conclusion:**

In the hypertensive patients, dexmedetomidine premedication provides better hemodynamic stability compared with midazolam, and because it decreases the antihypertensive requirements, its use might be beneficial.

**Trial registration:**

Trial registration: Clinicaltrials.gov identifier: NCT02058485.

## Background

Hypertension is the most common concomitant disease that we encounter in the practice of anesthesia. In hypertensive patients, excessive reduction in blood pressure is observed after anesthetic induction, while excessive increases in blood pressure are seen under stresses such as intubation, laryngoscopy, surgical incision and extubation. A decrease of greater than 20% in blood pressure can precipitates myocardial ischemia; decreases in diastolic blood pressure in particular can cause declines in both cerebral and myocardial perfusion. Elevations in blood pressure may cause myocardial ischemia and infarction by increasing cardiac work. Perioperative and postoperative complications in hypertensive patients are similar to those in normotensive patients [1–5]. However, in a study that evaluated 17,638 patients who had shown side effects such as hypotension and arrhythmias more frequently than not experienced major complications such as death and perioperative myocardial infarction in outpatient hypertensive procedures [6].

In the American College of Cardiology and the American Heart Association (ACC/AHA) guidelines for perioperative cardiovascular assessment, the usage of α-2 agonists, such as clonidine, is suggested in the treatment of perioperative hypertension, particularly in the presence of coronary disease. Furthermore, a limited number of studies have evaluated the usage of dexmedetomidine [7, 8]. Dexmedetomidine is 1600 times more selective to α-2 receptors than to α-1 receptors (clonidine, 200:1), and this high selectivity contributes to increases in hypnotic and analgesic efficacies and decreases in cardiovascular side effects [9, 10]. Potentially desirable effects include decreased requirements for other anesthetics and analgesics, a diminished sympathetic response to stress and the potential for cardioprotective effects against myocardial ischemia, along with minimal effects on respiration [11]. Currently, dexmedetomidine is being used in both the operating room and diagnostic and procedure units in adult patients for sedation and analgesic effects [12–16].

Some studies have compared dexmedetomidine versus midazolam for sedation in critical patients in the intensive care unit (ICU) setting [17]. However, we did not find a study comparing the usage of dexmedetomidine and midazolam for premedication in hypertensive patients in the literature. In our previous study comparing dexmedetomidine and midazolam for sedation during endoscopy, we observed more hemodynamic stability with dexmedetomidine, particularly in hypertensive patients [18]. Because of this observation, we designed this study with the hypothesis that the use of dexmedetomidine would provide better hemodynamic stability and would thus reduce the need for antihypertensive drugs in hypertensive patients for premedication during anesthetic management.

## Methods

Ethical approval for this study (Ethical Committee N° 2011/160) was provided by the Ethical Committee of Duzce University, Duzce, Turkey. After written informed consent from all patients was obtained, a total of 140 patients were included in our study between 10 January 2012 and 30 June 2013. The female patients included in this study were 40–60 years of age, with a body mass indices (BMIs) below 30 kg/m^2^, ASA I-II and were scheduled to undergo myomectomies or hysterectomies. Normotension was defined as a blood pressure below 140/90 mm Hg. Hypertensive patients had stage 1 hypertension (systolic blood pressure [SBP], 140–159 mmHg and diastolic blood pressure [DBP], 90–99 mmHg) or stage 2 hypertension (SBP; 160–179 mmHg and DBP; 100–109 mmHg) and were receiving antihypertensive therapy. We excluded patients who were recently diagnosed or were untreated; who were using angiotensin-converting enzyme (ACE) inhibitors as antihypertensive treatment; who had histories of myocardial infarction, heart block, heart failure, renal, pulmonary, cerebrovascular diseases or diabetes mellitus; who had difficult airways; whose time between the induction and beginning of surgery exceeded 15 min; and who required perioperative blood transfusions. This study was registered with ClinicalTrials.gov (NCT02058485).

### Study design

The normotensive and hypertensive patients were randomised into the 2 subgroups within themselves by a computer program: Group ND, normotensive patients who received dexmedetomidine; Group NM, normotensive patients who received midazolam; Group HD, hypertensive patients who received dexmedetomidine; Group HM, hypertensive patients who received midazolam before anesthesia. All patients were monitored by noninvasive blood pressure, 5-lead electrocardiography, pulse oximetry and bispectral index (BIS) using the same brand and model monitor (Datex-Ohmeda S/5 compact anesthesia monitor, GE Healthcare, Finland) that was being used in the operating room. The study drugs were prepared in 40 ml with 0.9% NaCl that was infused intravenously 15 min before anesthetic induction. Group ND received dexmedetomidine at a concentration of 0.5 μg.kg^−1^, Group NM received midazolam at a concentration of 0.025 μg.kg^−1^, Group HD received dexmedetomidine at a concentration of 0.5 μg.kg^−1^ and Group HM received midazolam at a concentration of 0.025 μg.kg^−1^. These drugs were calculated based on actual body weight and were prepared by a blinded anesthesiologist. Researchers, recorders and the attending anesthetist were blinded to the groups, and the medications were computer-selected from an unlabelled injector. For induction, 1 μg.kg^−1^ IV fentanyl was administered, and propofol was administered by continuous infusion (10 mg.kg.h^−1^) until the BIS value was 60, at which point the propofol was stopped. The total propofol amount for induction was recorded. Rocuronium at a concentration of 0.6 μg.kg^−1^ IV was administered, and endotracheal intubation was performed. Sevoflurane 1-2% in mixed air and O_2_ (50:50) was used as maintenance and was adjusted to maintain BIS values of 40–60. When the blood pressure increased by more than 25% over two consecutive measurements, nitro-glycerine infusion was initiated as an antihypertensive. Atropine was given as a 0.5 mg IV bolus for bradycardia (heart rate 45 beat/min) for at least 2 min. At the end of the surgery, sevoflurane was stopped, and the patients were extubated when they obeyed simple commands (eye opening, hand squeeze).

### Data collection

Systolic blood pressure (SBP), diastolic blood pressure (DBP), mean blood pressure (MBP) and heart rate (HR) values were recorded at the beginning (T_beginning_), 5 min after administration of the study drugs (T_preop 5 min_), 10 min after administration of the study drugs (T_preop 10 min_), immediately after induction of anesthesia (T_induction_), 1 min after intubation (T_intubation_), 5 min after intubation (T_intubation 5 min_), at the initial time of surgery (T_initial surgery_), 15 min after the start of surgery (T_surgery 15 min_), 30 min after the start of surgery (T_surgery 30 min_), 1 min after extubation (T_extubation_) and 5 min after extubation (T_extubation 5 min_). These values were primary outcomes measures. The amount of propofol necessary for induction, the time between induction and initial surgery, the requirement for antihypertensive therapy and the use of rescue atropine were recorded as secondary outcomes measures. Bradycardia, dry mouth and respiratory depression were evaluated as the side effects.

### Statistical analysis

Based on similar conducted studies, a change in heart rate by 10 beat/min had clinical significance in 4 groups [19]. Additionally, the pooled variance from the groups was determined to be approximately 12. Based on this information, the required sample size was calculated to be 30 for each group (total 120 patients) for a type I error of 0.05 with a statistical power of 80%. The groups were compared in terms of the patients’ clinical and demographic features, and the data were analysed with one-way variance analyses. To determine the categorical features of patients and their relationship to the group proper, *x*^2^ analyses (Likelihood Ratio or Pearson) were conducted. Because the initial measurements of hemodynamic parameters among the groups were significantly different, an adjustment was made by emitting the initial values from the values of the measurement period. After the adjustment, new values of the groups were compared by one-way variance analyses. Additionally, the separate periodic changes in the 4 groups were analysed by one-way repeated measures analysis of variance. A *P* < 0.05 was accepted as significant.

## Results

**Figure 1:**
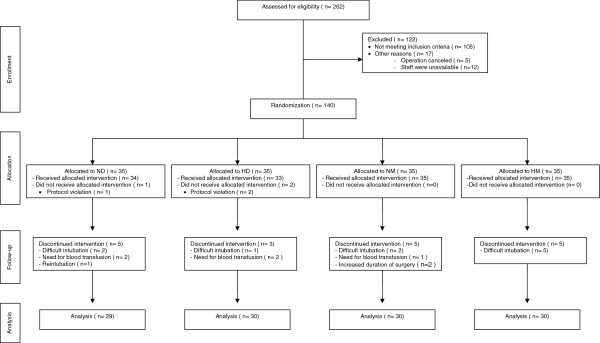
**CONSORT (Consolidated standards of reporting trials) flow diagram of the study.**

A total of 140 patients were enrolled, but only data from 119 patients were analysed in the study. (Group ND, n = 29; Group HD, n = 30; Group NM, n = 30; Group HM, n = 30) (Figure 1). There were no significant differences in age, weight, duration of operation and duration between induction and the start of surgery among the groups (Table 1). A comparison of ASA physical status revealed a significant difference among the groups (*P* = 0.006). The reason for this difference was that all of the patients in the hypertensive groups (Groups HD and HM) were assessed as ASA II, while some patients in the normotensive groups (Groups ND and NM) were assessed as ASA I.

**Table 1:** **Demographic and clinical data of the study**

	ND (***n*** = 29)	HD (***n*** = 30)	NM (***n*** = 30)	HM (***n*** = 30)	p
**Age (yrs)**	46 ± 5	49 ± 6	48 ± 6	46 ± 6	0.386
**Weight (kg)**	72 ± 11	79 ± 10	74 ± 11	77 ± 11	0.087
**ASA (I/II) (** ***n*** **)**	7/ 22	0/30	5/25	0/30	0.002^*^
**Stage of hypertension (1/2) (** ***n*** **)**	-	21/9	-	18/12	0.261
**Duration of operation (min)**	92 ± 25	87 ± 26	81 ± 22	92 ± 22	0.248
**Duration of induction-surgery (min)**	17 ± 8	14 ± 7	13 ± 4	14 ± 4	0.096
**Propofol amount (mg)**	86 ± 27	102 ± 34	95 ± 40	114 ± 44	0.036^*^
**Rescue antihypertensive (%)**	0	0	3	17	0.007^*^

Values are displayed as means ± standard deviations, numbers, and percentages. *p < 0.05 compared between groups.

The amount of propofol required for induction was significantly different among the groups (*P* = 0.036). The propofol requirements were highest in Group HM and lowest in Group ND.

The perioperative requirement for antihypertensive drugs was significantly higher in Group HM (*P* = 0.007) (Table 1).

Regarding side effects, dry mouth was observed most commonly in Group HD, followed by Group ND (*P* < 0.001). The incidence of bradycardia was highest in Group HD, followed by Group ND (*P* < 0.001). There was no significant difference in atropine usage among the groups (*P* = 0.530). Respiratory depression was not observed (Table 2).

**Table 2:** **Incidence of side effects**

	ND (***n*** = 29)	HD (***n*** = 30)	NM (***n*** = 30)	HM (***n*** = 30)	p
**Dry mouth [n (%)]**	7 (24)_a,b_	12 (40)_b_	2 (7)_a_	1 (3)_a_	<0.001^*^
**Bradycardia [n (%)]**	1 (38)_a,b_	18 (60)_b_	3 (10)_a_	4 (13)_a_	<0.001^*^
**Respiratory depression [n (%)]**	0 (0)_a_	0 (0)_a_	0 (0)_a_	0 (0)_a_	1
**Rescue atropine [n (%)]**	3 (10)_a_	4 (13)_a_	1 (3)_a_	2 (7)_a_	0.503

Values are displayed as percentages. *P < 0.05 compared between groups. In each row, same letters near the proportions were shown not statisticaly significant differences between groups.

### Comparison of hemodynamic data

There were significant differences in the initial values of SBP, DBP and MBP (T_beginning_) among the groups. These differences were due to the hypertensive and normotensive groups. The hemodynamic comparisons were performed after these initial differences were removed statistically.

**Figure 2:**
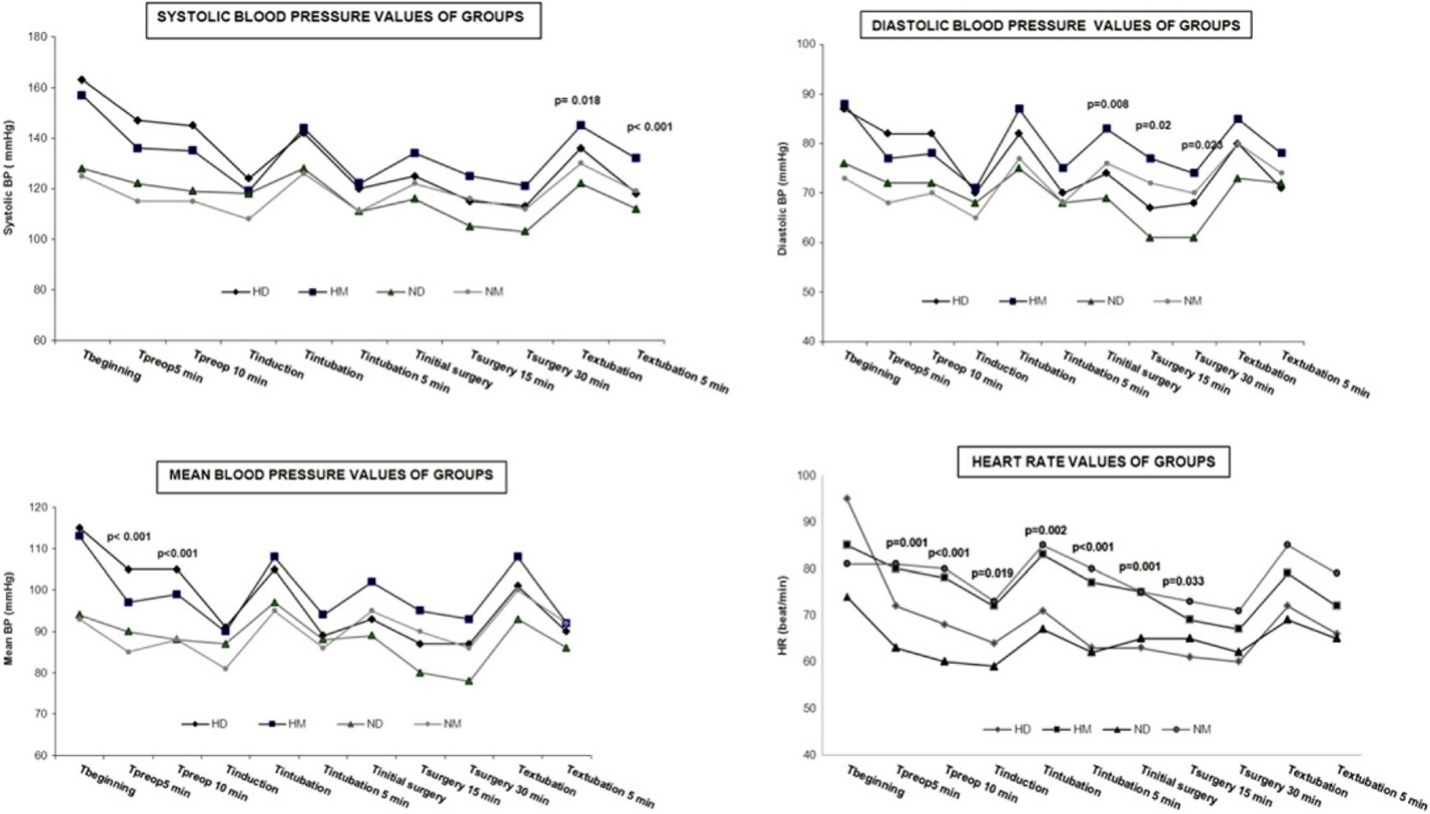
**Hemodynamic means of groups.**

SBP was significantly different between the normotensive and hypertensive groups at the following time points: premedication 5th min (T_preop 5 min,_*P* < 0.001), premedication 10th min (T_preop 10 min_, *P* = 0.002), induction (T_induction_, *P* < 0.001), intubation 1st min (T_intubation_, *P* < 0.001), intubation 5th min (T_intubation__5 min_, *P <* 0.001) and initial surgery (T_initial surgery_, *P* < 0.001). The mean values of SBP at T_preop 5 min_, T_preop 10 min_ and T_induction_ were significantly reduced in the hypertensive groups. In particular, a marked reduction was noted in Group HM after induction. The mean values of SBP at T_intubation_, T_intubation 5 min_ and T_initial surgery_ were significantly increased in the normotensive groups, particularly in Group NM. There were significant differences at 15 min of surgery (T_surgery 15 min_) and 30 min of surgery (T_surgery 30 min_) between the normotensive and hypertensive groups, but no significance between the hypertensive groups. Nevertheless, there were significant differences at 1 min after extubation (T_extubation_) and 5 min after extubation (T_extubation 5 min_) between Groups HD and HM. (T_extubation,_*P* = 0.018 and T_extubation 5 min,_*P* < 0.001). Considering the averages, a minimum increase was observed in Group HD (Figure 2).

DBP values did not differ significantly between the groups at the different time points. However, the mean value of DBP at T_preop 5 min_ was significantly decreased in Group HM (HM-ND *P* = 0.005; HM-NM *P* = 0.023; HM-HD *P* = 0.029). In particular, a marked reduction was noted after induction (T_induction_) in the hypertensive groups (HD-ND *P* = 0.008; HD-NM *P* = 0.02; HM-ND *P* = 0.009; HM-NM *P* = 0.023). The values were significantly higher in Group NM at T_initial surgery_, T_surgery 15 min_ and T_surgery 30 min_ (Figure 2).

MBP significantly decreased in Group HM at T_preop 5 min_ and T_preop 10 min_. In addition, MBP was significantly lower in the hypertensive groups at the other time points (T_induction_, T_intubation_, T_intubation 5 min_, T_initial surgery_, T_surgery 15 min_, T_surgery 30 min_, T_extubation_ and T_extubation 5 min_) (Figure 2).

There was a significant difference in the initial values of HR (T_beginning_) in Group ND (*P* = 0.009). The evaluation was performed after this initial difference was removed statistically. HR significantly decreased in the dexmedetomidine groups (Groups HD and ND), at the premedication time points (T_preop 5 min_ and T_preop 10 min_). When the mean values were compared, Group HD had the largest decrease. Additionally, there were significant reductions in HR at T_induction_, T_intubation_, T_intubation 5 min_, T_initial surgery_, T_surgery 15 min_, T_surgery 30 min_, T_extubation_ and T_extubation 5 min_ in Group HD (Figure 2).

## Discussion

At the measured time points (after premedication, induction of anesthesia intubation, early surgical period and extubation) hypertensive patients showed significant changes compared to normotensive patients. There was no difference between dexmedetomidine and midazolam use. However, the requirement for perioperative antihypertensives was significantly higher in patients who received midazolam. Because antihypertensives were used in this group that interfered with blood pressure values, the values were reduced to the blood pressure values of patients that were treated with dexmedetomidine; therefore, it is possible that significant differences may have been eliminated. If perioperative antihypertensive treatment had not been administered in the hypertensive midazolam patients, the data may have indicated a statistically significant difference when compared to patients who were given dexmedetomidine.

It is well known that perioperative hemodynamic fluctuations are observed more often in hypertensive patients than in normotensive patients [20]. These fluctuations can occur for the duration of anesthesia and are well tolerated in healthy individuals; however, these pressure changes and increased sympathetic activity can have harmful effects on hypertensive patients [21].

Ghignone et al. compared clonidine and diazepam as premedications for normotensive and hypertensive patients and found that clonidine was more effective in preventing reflex tachycardia caused by laryngoscopy and endotracheal intubation, and they reported that intraoperative fluctuations decreased [10]. Singh et al. showed that oral clonidine as a premedication provided more stable hemodynamics compared to placebo (in this study, hypertension was not included in the study) [22]. Mariappan et al. showed similar hemodynamic profiles when they compared oral clonidine premedication and perioperative dexmedetomidine infusion [23]. Most studies showed that, compared to saline, dexmedetomidine as a premedication significantly decreased hypertensive and tachycardia responses that occur due to intubation [8, 24–26]. Becker et al. reported that the dexmedetomidine infusion after intubation in craniotomy patients resulted in fewer antihypertensive requirements [27]. These studies were conducted in normotensive patients. A study comparing dexmedetomidine and three different doses of midazolam showed that dexmedetomidine decreased mean blood pressure and HR compared to all of the different midazolam dosages [28]. Nevertheless, in all studies, hypertensive patients were either excluded or mixed with normotensive patients. In our study, it was established that hemodynamic fluctuations were more common in hypertensive patients than in normotensive patients. Furthermore, we observed that the hemodynamic response was more distinctive in hypertensive patients who received midazolam as premedication, and the data indicated that in the same group, the use of antihypertensive drugs was more common.

Another parameter that we evaluated was the amount of propofol used during induction in the different groups. For this purpose, we used a propofol infusion and BIS monitoring and stopped the infusion when BIS reached 60. In the hypertensive groups, the propofol requirement was higher, while in the dexmedetomidine groups, a significant decrease in the requirement was observed. Studies have shown that the perioperative and preoperative usage of dexmedetomidine decreases the requirement for anesthetics [24, 25, 29–32]. In these studies, dexmedetomidine was compared only with saline. The reducing effects of α-2 agonists on sympathetic neural activity and catecholamine in circulation are responsible for the decrease in anesthetic requirement [33]. In addition, it is thought that α-2 agonists inhibit ion conduction through types L and P Ca channels in the central nervous system and stimulate K channels activated with voltage-related Ca. Other sedatives have GABAergic effects that are different from the hypnotic effects of dexmedetomidine.

In our study, the incidence of bradycardia was significantly higher in the dexmedetomidine groups. This difference was especially pronounced in the hypertensive patients. However, the usage of rescue atropine was not significantly different. Previous studies on the use of dexmedetomidine in hypertensive patients did not specify heart rate characteristics [10, 34]. Nevertheless, in the study by Wallace et al., the frequency of bradycardia (<40 beats/min in the study) was reported to be 10% in the cardiovascular patients who had been premedicated with clonidine [35]. In our study, we accepted bradycardia at <45 beats/min; under this condition, the bradycardia frequencies were 60% in hypertensive patients and 38% in normotensive patients who were treated with dexmedetomidine.

It is known that dexmedetomidine increases the frequency of dry mouth due to the drug’s inhibitory effect on salivation. In our study, dry mouth was also encountered more commonly in patients who were treated with dexmedetomidine, particularly Group HD.

During the planning of this study, we chose gynaecologic operations to decrease hemodynamic changes caused by a variety of surgical stimulations and gender. However, this choice excluded the evaluation of hemodynamic changes in male patients, which is one of the limitations of this study.

## Conclusions

Based on our study, dexmedetomidine as a premedication seems to be a better choice than midazolam for hypertensive patients. In addition, dexmedetomidine may have the benefit of decreasing anesthetic requirements. Nonetheless, it should be taken into consideration that dexmedetomidine increases the incidence of dry mouth and bradycardia in hypertensive patients.

## Abbreviations

IV: Intravenous
SBP: Systolic blood pressure
DBP: Diastolic blood pressure
MBP: Mean blood pressure
HR: Heart rate
T_beginning_: Baseline measurement
T_preop5 min_: Measurement time of 5 minutes after administration of study drugs
T_preop 10 min_: Measurement time of 10 minutes after administration of study drugs
T_induction_: Measurement time of immediately after induction
T_intubation_: Measurement time of 1 minute after intubation
T_intubation__5 min_: Measurement time of 5 minute after intubation
T_initial surgery_: Measurement time at surgical incision
T_surgery 15 min_: Measurement time of 15 minutes after surgical incision
T_surgery 30 min_: Measurement time of 30 minutes after surgical incision
T_extubation_: Measurement time of 1 minute after extubation
T_extubation 5 min_: Measurement time of 5 minute after extubation
ACC/AHA: American College of Cardiology and the American Heart Association
ICU: Intensive care unit
BMI: Body mass indices
ACE: Angiotensin-converting enzyme
BIS: Bispectral index.
